# Resurgent Syphilis Across the Globe: A Public Health Perspective on Bridging Surveillance and Strategy

**DOI:** 10.3390/pathogens14111148

**Published:** 2025-11-12

**Authors:** Jorge Luis Espinoza, Ly Quoc Trung

**Affiliations:** 1 Faculty of Health Sciences, Kanazawa University, Kanazawa 920-0942, Ishikawa, Japan; 2 Soctrang Hospital for Women and Children, Soc Trang 950000, Vietnam; lqtrung@bvchuyenkhoasannhist.com.vn

**Keywords:** syphilis resurgence, sexual health, congenital syphilis, health equity, surveillance systems

## Abstract

Syphilis, a curable sexually transmitted infection, has resurged globally, challenging public health systems in both high-income countries and low- and middle-income countries (LMICs). In nations like the United States, the United Kingdom, parts of Europe, Canada, and Japan, cases have surged due to declining condom use, digital platforms facilitating casual sex, and practices like chemsex and broader drug use for sex, with rising congenital syphilis rates. In LMICs, such as those in East Africa, South Asia, Latin America, and Southeast Asia, limited healthcare access, inadequate prenatal screening, and socioeconomic barriers drive persistent high prevalence, particularly among pregnant women and vulnerable populations. Despite contextual differences, shared drivers include stigma, health disparities, and outdated surveillance systems. This resurgence underscores the need for globally coordinated, equity-focused strategies, including universal syphilis testing, modernized surveillance, and context-specific sexual health education. Addressing structural and behavioral factors through collaborative international efforts is critical to reversing this trend and strengthening global STI control.

## 1. Methods for Reference Identification

References were identified through a combination of manual searches in PubMed, Google Scholar, and Web of Science, complemented by consultation of open-access surveillance databases from WHO, CDC, ECDC, and national public health agencies. Relevant publications were also identified through citation tracking and expert recommendation to ensure inclusion of up-to-date global data and region-specific reports not indexed in standard databases.

## 2. Perspective

Although the discovery of penicillin dramatically reduced syphilis-related morbidity and mortality, the infection has never been fully controlled. Over time, periodic resurgences have occurred worldwide, particularly among key risk populations. Syphilis has reemerged as a global public health threat. Across both high-income and low- and middle-income countries (LMICs), the disease has surged in recent years. The reasons for this rise are multifaceted and context-specific yet globally interconnected. The scale and distribution of new cases, especially among vulnerable and underserved populations, highlight systemic failures in sexual health education, diagnostic outreach, surveillance infrastructure, and equitable care.

In high-income countries such as the United States, the United Kingdom, and Japan, syphilis has defied decades of progress in STI control. In the United States, cases have tripled since 2010, reaching their highest numbers since the 1950s, with particularly sharp increases among men who have sex with men (MSM), as well as growing incidence among heterosexual individuals and newborns. The UK has seen a similar trend, with rising rates among MSM and an increasing proportion of cases among heterosexuals [1]. Japan presents a particularly striking case: syphilis cases rose from 577 in 2010 to over 14,000 in 2024, with a shift from MSM-driven to heterosexual transmission, especially among young women [2]. Multiple behavioral and structural factors underpin this resurgence. The adoption of HIV pre-exposure prophylaxis (PrEP) has contributed to a decline in condom use, especially among MSM. Digital platforms and dating apps have facilitated casual and anonymous sexual encounters, creating dense and shifting sexual networks. Practices such as chemsex—where sexual activity is combined with stimulant drug use—have further fueled transmission [3]. Furthermore, beyond the specific context of chemsex, the broader practice of using drugs to facilitate or enhance sexual activity (“drug use for sex”) is recognized as a significant risk factor for syphilis transmission, creating environments of disinhibition and prolonged sexual risk-taking. Surveillance systems have not kept pace with these evolving risk environments, and stigma continues to discourage testing and treatment [4]. This trend is mirrored in other high-income regions. Surveillance data from the European Centre for Disease Prevention and Control (ECDC) reveal a steady increase in syphilis cases across the European Economic Area, with notable rises in countries like Germany, Ireland, and Malta, primarily driven by transmission among MSM (https://www.scribd.com/document/880780199/Syph-Aer-2023-Report, accessed on 25 October 2025). Similarly, Canada has reported a dramatic resurgence, with infectious syphilis rates increasing over 10-fold since 2000, alongside a parallel crisis of congenital syphilis [5]. Australia also faces a significant, persistent syphilis burden, with complex epidemiology involving both key populations and the general population [6]. The reported incidence of syphilis in China escalated from 4.50 per 100,000 in 2003 to 34.04 per 100,000 in 2021, making syphilis the most commonly reported STI in mainland China [7].

**Figure 1:**
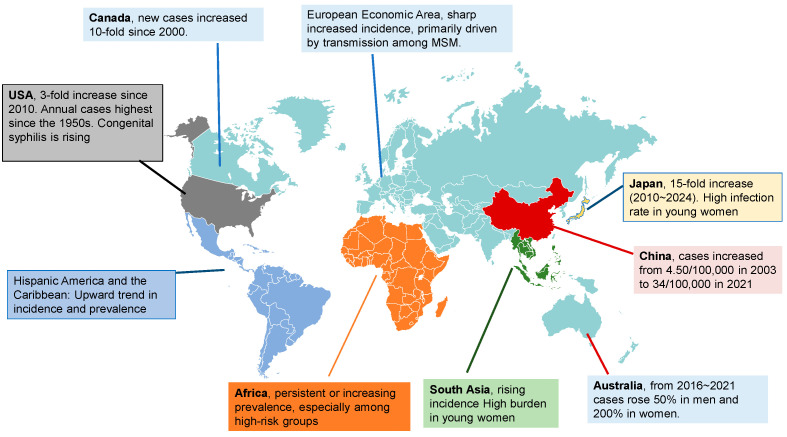
Global Hotspots of Syphilis Resurgence. Geographic regions with rising or sustained high syphilis burden are highlighted. The determinants of resurgence vary by region, as discussed in the main text.

However, syphilis resurgence is by no means confined to industrialized nations. Recent epidemiological analyses reveal sustained or rising syphilis incidence in many LMICs. In East Africa, countries such as Ethiopia, Tanzania, Malawi, and Rwanda report elevated prevalence, particularly among pregnant women, sex workers, and people living with HIV, while the Central African Republic and Equatorial Guinea are consistently identified as having among the world’s highest syphilis-related disease burdens [8,9,10]. Similar trends are observed in Latin America and the Caribbean, where access to prenatal screening and diagnostics, combined with systemic health infrastructure challenges, have allowed syphilis—and congenital syphilis in particular—to persist at high levels [11]. In South Asia, India bears a disproportionately high burden, accounting for a substantial portion of global syphilis cases, with high prevalence among pregnant women and key populations, though surveillance remains challenging [12]. In Southeast Asia, syphilis remains a significant public health concern across the region, with particular vulnerability among women of reproductive age and ongoing risks for congenital syphilis due to incomplete screening and systemic health challenges [13] (Figure 1).

Notably, China demonstrated measurable success in reducing congenital syphilis following the implementation of national screening and treatment programs between 2011 and 2018 [14]. Similar targeted antenatal screening interventions in Cuba and India have shown that sustained prevention programs can reverse adverse trends [15,16].

Despite differences in context, there are overlapping drivers of syphilis resurgence across both income settings. In LMICs, limited access to healthcare—including diagnostics, treatment, and antenatal screening—is a major barrier to STI control. Socioeconomic factors such as poverty, health illiteracy, and stigma further impede prevention efforts. In high-income countries, while services are more accessible in theory, persistent disparities, declining condom use, and unregulated digital platforms have eroded prevention gains. In high-income countries, MSM, sex workers, older adults, and migrants in transit represent key risk groups. Migrants in transit are particularly vulnerable due to limited access to healthcare, exposure to risk factors during displacement, and their potential role in sustaining transmission chains across borders [11].

The global resurgence of syphilis signals not only sexual health policy failures but broader public health system weaknesses. This curable infection persists in advanced and resource-limited settings alike, serving as both warning and opportunity for STI and reproductive health models emphasizing equity, surveillance, and early intervention [17]. Reversing trends demands coordinated, equity-focused global strategies: universal syphilis testing access, especially for pregnant women; integration into routine antenatal care; and expanded point-of-care diagnostics to curb congenital cases. Modernize surveillance with real-time digital reporting, private sector data integration, and anonymized platform analytics for better outbreak detection. Reframe STI control via global solidarity: Japan’s resurgence despite infrastructure shows prevention dismantling risks; East Africa/Latin America’s burden reveals absent systems. Both are unacceptable. The fight against syphilis’s resurgence cannot be won in isolation. As a disease that crosses borders with ease, international collaboration is paramount for sharing data, coordinating research, and ensuring a unified global response. However, this global effort must be informed by regional expertise; local health workers are indispensable for crafting culturally sensitive interventions and building trust within affected communities. Ultimately, any strategy must be guided by a principle of health equity, actively targeting the systemic barriers that make vulnerable populations bear the greatest burden of this disease. While specific tactics for combating syphilis may vary between high- and low-income countries, any sustainable solution must be rooted in this shared commitment (Table 1).

Importantly, epidemiological data must be interpreted with caution. The COVID-19 pandemic disrupted surveillance systems, reduced testing rates, and delayed case notifications, leading to transient underestimation of incidence in several regions. Moreover, prevention gaps—including shortages of benzathine penicillin, misuse of alternative antibiotics, and uneven access to injectable formulations—continue to limit effective case management, even in high-income countries such as Japan. These challenges highlight persistent inequities in prevention and treatment access globally.

## 3. Conclusions

The global resurgence of syphilis offers an urgent reminder: progress in infectious disease control is never permanent and can be reversed without vigilance, investment, and innovation. Furthermore, sustaining this effort requires continued investment in fundamental research, including biomolecular studies of Treponema pallidum to elucidate pathogenesis and antigenic variation, which is crucial for the development of an effective vaccine. The tools to curb syphilis exist—but they must be deployed strategically, equitably, and with the full weight of global public health behind them.

## Figures and Tables

**Table 1:** Key differences in syphilis risk factors and structural challenges between high-income and low-/middle-income countries.

Factor	High-Income Countries	Low-/Middle-Income Countries	Potential Solutions
Primary Risk Groups	MSM, sex workers, older adults, migrants in transit	Pregnant women, general population, sex workers	Provide targeted education, counseling, and support services for sex workers and other high-risk groups
Access to Care	Generally good, but with gaps for marginalized groups	Often limited, especially in rural and low-resource areas	Expand community-based clinics and mobile STI services in underserved areas
Condom Use	Declining, especially with PrEP users and in casual encounters	Low due to cost, stigma, or lack of education	Revitalize condom promotion campaigns tailored to digital-age populations and key groups
Digital/Tech Influence	High (dating apps, chemsex, anonymous encounters)	Moderate but increasing in urban centers	Leverage social media and digital platforms for sexual health education and partner notification tools
Prenatal Screening	Moderate to high, but coverage gaps exist	Often poor, contributing to high congenital syphilis rates	Ensure universal access to antenatal syphilis screening with rapid point-of-care tests
Health System Challenges	Funding cuts, outdated surveillance, stigma	Under-resourced systems, workforce shortages, stigma	Improve integration of STI services into primary care and increase health workforce capacity
Health System Shocks (e.g., Pandemics)	Reduced STI screening and disrupted services	Severely disrupted services and intensified health inequities	Strengthen public health education and build resilient, adaptive health systems

Abbreviations: MSM: men who have sex with men; PrEP: pre-exposure prophylaxis; STI: sexual transmitted infections.

## Data Availability

No new data were created or analyzed in this study.
